# Hydrodynamic Clustering of Human Sperm in Viscoelastic Fluids

**DOI:** 10.1038/s41598-018-33584-8

**Published:** 2018-10-22

**Authors:** Kenta Ishimoto, Eamonn A. Gaffney

**Affiliations:** 1 Wolfson Centre for Mathematical Biology, Mathematical Institute, University of Oxford, Oxford, OX2 6GG UK; 2 Graduate School of Mathematical Sciences, The University of Tokyo, Tokyo, 153-8914 Japan

## Abstract

We have numerically investigated sperm clustering behaviours, modelling cells as superpositions of regularised flow singularities, coarse-grained from experimentally obtained digital microscopy of human sperm, both in watery medium and a highly viscous–weakly elastic, methylcellulose medium. We find that the cell yaw and cell pulling dynamics inhibit clustering in low viscosity media. In contrast clustering is readily visible in simulations modelling sperm within a methylcellulose medium, in line with previous observations that bovine sperm clustering is much more prominent in a rheological polyacrylamide medium. Furthermore, the fine-scale details of sperm flagellar movement substantially impact large-scale collective behaviours, further motivating the need for the digital microscopy and characterization of sperm to understand their dynamics.

## Introduction

The scale of inefficiency for the male gamete in mammalian fertilization is staggering: a fertile human ejaculate averages around 180 million sperm^1^, yet in almost all circumstances no more than one cell from this population fertilises an egg. With such high numbers of sperm, collective behaviours are invariably apparent, as perhaps first reported in terms of wave-like patterns in concentrated bull sperm suspensions during an investigation of artificial insemination by Rothschild in the 1940s^2^.

**Figure 1:**
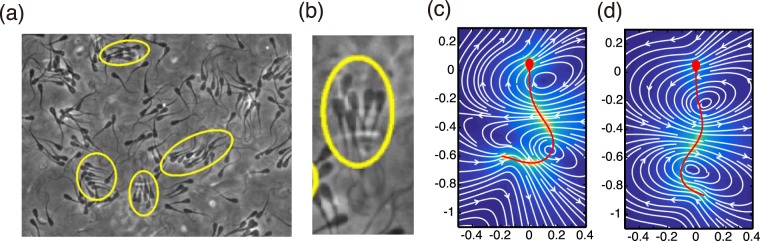
(**a**) Dynamic clustering for bovine sperm in 1% long chain polyacrylamide, reproduced from^4^ with permission via the creative commons license, http://creativecommons.org/licenses/by/4.0/. (**b**) A blow up from (**a**), in turn reproduced from Tung *et al*.^4^, with permission, highlighting that sperm flagella are not necessarily synchronised within a cluster. The predicted fluid flow around a human sperm in a low viscosity, watery Newtonian medium (LVM, (**c**)), with dissolved components such as glucose and physiological ions for cell maintenance, together with albumin to prevent sperm-glass adhesion^9^. The analogous predicted fluid flow for a human sperm swimming in a highly viscous–weakly elastic 1% methylcellulose solution (HVM, (**d**)), which increased viscosity to about 140 times that of water, which is roughly the average of human female reproductive tract mid-cycle mucus^9,48^. The predicted flow field is an instantaneous snap shot, with axes labels in units of flagellum length, and with the sperm in red, the velocity magnitude given by the intensity of blue shading and the streamlines in white. The data used to generate plots (**c**,**d**) may be found in the references^35,36^.

Consequently, collective effects occur in mammalian sperm handling and more generally are anticipated in the early stages of the sperm journey to the egg^3^, which includes propagation through the highly rheological mucus of the cervix. Hence collective behaviours in viscoelastic media are also highly relevant physiologically and it has recently been reported that viscoelasticity induces a dynamic and fluctuating bovine sperm clustering, in which cluster members are continuously exchanging^4^, as illustrated in Fig. 1(a,b). Thus our fundamental aim is to develop a modelling framework to explore how and why properties of the surrounding medium influences sperm clustering behaviour.

One should also note that the bovine collective behaviour is quite distinct from rodent sperm trains, which involve sperm-sperm attachments^5^. The absence of attachments for the fluctuating clustering of bovine sperm in rheological media is more suggestive of hydrodynamic interactions which, due to the low Reynolds number of the flow, can significantly affect collective behaviours^6,7^. Furthermore, it is well known that the sperm flagellum beat pattern is distinctively different between Newtonian media and either highly viscous–weakly elastic, or highly viscoelastic media^8–10^. Thus, the hydrodynamic interactions between sperm differ on comparing swimming in Newtonian and rheological fluids not only due to the different media, but also due to the different flagellum waveforms^11^.

Hence examining the collective behaviour of sperm clustering requires a multi-scale modelling framework that upscales the detailed flagellar waveform dynamics. However, sperm-swimming simulations have generally only been considered at the level of the individual cell^12–14^, with the notable exception of a recent large direct numerical simulation^15^, while other population studies have considered active colloidal particle populations^16–18^ or bacterial flagellates^19^. Furthermore, these population level investigations have either directly numerically simulated the population, averaged the individual dynamics over the propulsion cycle, or considered a coarser, mean field, approximation^15–23^. For the current context of this study, a refined upscaling – in particular incorporating the fast timescale associated with the swimmer propulsion cycle, i.e. the flagellum beat cycle for sperm – is developed while avoiding the computational cost of direct numerical simulation for all the swimmers.

A further aspect of sperm swimming in extremely close proximity is the prospect that the hydrodynamics couples with the solid filament mechanics to induce flagellar synchronisation^24^. However, we do not resolve this even finer level of detail in our study. In particular, the fluid-structure interactions require elastohydrodynamic simulations^15,25–30^ together with a resolution of molecular motor contractions within the flagellum^31–34^, though the latter is currently not feasible with elastohydrodynamic predictions. Furthermore, flagellar sychronisation is not observed to be necessary for clustering, as illustrated in Fig. 1(b). In turn, we therefore focus on experimental digital video microscopy for the flagellum waveforms, rather than attempting to predict the beat patterns *ab initio*.

Hence our scope is limited to scenarios where the sperm flagellar waveform has been digitised and we proceed in developing a modelling framework by exploiting previous studies, where the flagellar waveform data has been captured and further simplified using data analytic techniques, in particular principal component analysis (PCA). This has been then used to computationally determine the flow field around a sperm, a snapshot of which is illustrated for a water-like, low viscosity, Newtonian medium, referred to as LVM below, in Fig. 1(c) and for a highly viscous–weakly elastic medium of 1% methylcellulose solution, referred to as HVM below, in Fig. 1(d). These flow fields are complex, and hence PCA is further utilised to enable an accurate simplification incorporating near-field information about the swimmer. These PCA flow field representations may be further, but accurately, simplified in terms of a small number regularised Stokeslets. This is especially useful as regularised Stokeslet representations have a straightforward physical interpretation, in contrast to PCA expansions, while capturing most of the variation of the flow field^35,36^ and ultimately allowing a novel upscaling of individual dynamics and physics into larger scale, population models.

Consequently, our detailed objective is to use such PCA-derived coarse-graining representations of the sperm flow fields to construct a multi-scale model of sperm collective behaviour, which assimilate experimental flagellar waveforms, to explore dynamic sperm cell clustering. In addition to examining whether the full temporal resolution of high-speed digital microscopy is required for coarse graining, a further objective will also be to examine how differences at the individual cell level influence the tendency of sperm to dynamically cluster. We will also briefly compare our predictions with observations of bull sperm in both a low viscosity medium and viscoelastic polyacrylamide, though acknowledging that this study does not capture a number of the experimental details.

## Results

### Regularised Stokeslet representations

The characteristic waveform in both the LVM and HVM media is extracted from high-speed digital microscopy images and simplified using PCA, which highlights that the waveform is well approximated via a limit cycle within the phase space of its first few PCA modes and thus has an associated limit cycle phase, denoted *ϕ*(*t*) below^35,36^. The associated flow field surrounding the sperm is calculated using the PCA waveform as a boundary condition of the inertialess fluid equations. These in turn are solved via the boundary element method, assuming the fluid flow obeys the Newtonian Stokes equations for LVM and linear Maxwell equations for HVM (Fig. 1(b,c) and citations^35–37^), with further details provided in the Methods section.

**Figure 2:**
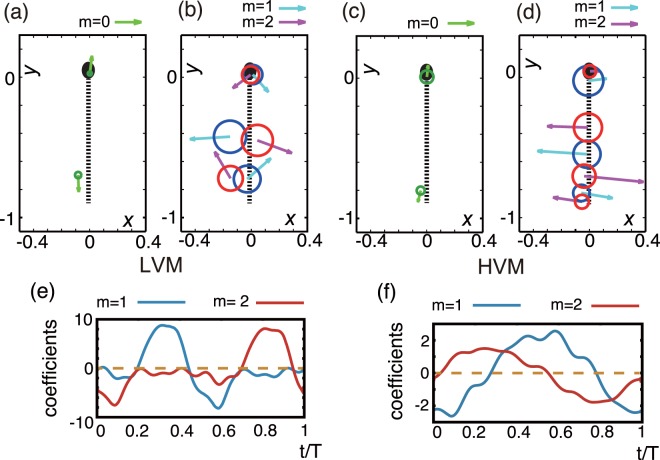
The regularised Stokeslet representation for a swimming human sperm in a low viscosity, watery Newtonian medium (LVM, (**a**,**b**)) and a highly-viscous, weakly elastic, 1% methylcellulose solution (HVM, (**c**,**d**)), with axes labels in units of flagellum length. The regularised Stokeslet representations for the time-averaged flow field ((**a**,**c**), *m* = 0) and for the lowest two flow bases ((**b**,**d**), *m* ∈ {1, 2}) are shown. The origin, length and direction of the arrows give the location, magnitude and direction of the force singularities, and the circle radius corresponds to the regularised parameter. The dashed lines show the centreline of the model sperm, while the black ellipse shows the location of the sperm head for illustration. The time dependent weightings for modes 1, 2 for LVM are plotted for time *t* over a flagellum beat pattern period, *T*, in (**e**) with analogous plots for HVM in plot (**f**). Note that modes 1, 2 are out of phase so that the regularised singularities in plots (**b**,**d**) do not simply undergo constructive or destructive interference. The data used to generate plots (**b**,**d**,**e**,**f**) may be found in^35,36^, together with further details on the derivation of the representation.

Subsequently applying the PCA method to the calculated flow field, denoted flow PCA below, has also revealed that the time-varying flow fields can be well approximated by the linear superposition of small numbers of regularised Stokeslets^38^, with time-dependent weightings as reported in previous work^35,36^, and summarised in the paragraphs below. In particular, to represent the velocity fields resulting from flow PCA in terms of regularised Stokeslets, we firstly consider the beat period averaged flow, labelled by *m* = 0, together with the first *M* PCA flow modes. The velocity fields associated with these are approximated via *L*(*m*) regularised singularities, with the mode label *m* ∈ {0, …, *M*}, as summarised in Fig. 2 for *M* = 2, *m* ∈ {0, 1, 2}. With the singularity label *l* ∈ {1, 2, … *L*(*m*)}, where *L*(*m*) denotes the number of singularities associated with mode *m*, each of these singularities is located at ${{\boldsymbol{x}}}_{0}^{(m,l)}$, with regularization parameter *ε*^(*m*,*l*)^.

In practice the number of PCA modes is chosen to be *M* = 2 throughout this study, representing a balance between capturing the variation of the fluid flow and keeping the flow representation simple, with the choice of *L*(*m*) reflecting the same tension. This tension is analysed in detail in^35,36^, with *M* = 2 modes capturing 68% of the variation for LVM and 90% for HVM. The number of singularities chosen to represent each mode, *L*(*m*), is such that on optimising for singularity location, size, direction and regularisation parameter only a further 7% of the variation is lost in either the LVM or the HVM cases while keeping the number of singularities small. The resulting singularities therefore, while manageable in number, also capture the main details of the fluid flow and are explicitly shown in plots 2(a,b) for the LVM case with ${{\boldsymbol{x}}}_{0}^{(m,l)}$ located at the base of each arrow. The magnitude and direction of the regularised Stokeslet are given by the length and direction of the depicted arrow, while the radius of the circle centred at the arrow base gives the regularisation parameter. Analogous plots for the HVM case are given in plots 2(c,d).

Letting *i*, *j* ∈ {1, 2, 3} label spatial dimension, with the regularised singularity given by ${G}_{ij}({\boldsymbol{x}},{{\boldsymbol{x}}}_{0}^{(m,l)};\varepsilon )$^38^ the flow is therefore approximated by^35,36^

$${\boldsymbol{u}}({\boldsymbol{x}},t)=\sum _{m=0}^{M}\,\sum _{l=1}^{L(m)}\,\sum _{j=1}^{3}\,{G}_{ij}({\boldsymbol{x}},{{\boldsymbol{x}}}_{0}^{(m,l)};{\varepsilon }^{(m,l)}){f}_{j}^{(m)}(\varphi (t)).$$

Note that the zero mode force strengths ***f***^(0)^ are independent of time with a magnitude and direction given by the arrows of plots 2(a,c) for the LVM and HVM cases, respectively. The remaining force strengths ***f***^(*m*)^ for the LVM case are given by their respective magnitudes and directions, as depicted by the arrow lengths of plot 2(b), multiplied by a phase factor, which is a function of the limit cycle phase *ϕ*(*t*), obtained from the flagellar PCA. The phase factors are functions of time and also the mode label *m*, but not the singualrity label, *l*, with the latter independence following as all singualrities approximatig a flow field PCA mode inherit the temporal modulation of the PCA mode. These phase factors are explicitly plotted over a flagellar beat period, *T*, in Fig. 2(e) for *m* = 1, 2 and analogous remarks apply for the HVM cases with plots 2(d,f), while additional details have been extensively described previously^35,36^.

In both media, the time-averaged flow fields associated with *m* = 0 are accurately represented by a pusher-type swimmer, with two regularised Stokeslets (Fig. 2(a,c)). However, there are also notable and distinct differences with rheology (Fig. 2(b,d)). The time-dependent flow in the LVM case is characterised by large lateral forces, together with counter-forces at the sperm head and distal flagellum, with the force coefficients ${f}_{j}^{(m)}$, *m* ∈ {1, 2}, switching on and off, with changing signs during the beat cycle. This in turn is associated with an overall flow field that switches between pusher- and puller-type profiles during the flagellum beat. In turn this oscillatory dynamics induces an extensive cell yaw, that is oscillatory lateral movement relative to the overall progressive direction of the cell^35^. In contrast, for the HVM case, large viscous resistance appears to suppress cell yaw, and the time-resolved flow field is well described by travelling waves of lateral forces^36^, with a flow profile that is always of pusher-type^36^ throughout the beat period. These regularised Stokeslet representations motivate the modelling of a sperm via objects composed of $K={\sum }_{m=0}^{M}\,L(m)$ regularised Stokeslets, in turn approximately representing *K* spheres, as briefly discussed in the Methods section.

### Modelling collective behaviour

To consider collective behaviour, we firstly use the above regularised Stokeslet superposition as a representation for the flow induced by each sperm, with all sperm swimming in a 2D-plane, noting that sperm cells accumulate adjacent to a flat surface^39^. The flow is still well approximated even in the presence of the wall, given the typically observed scale of ≈15*μ*m for the sperm-substrate separation^9,35^, s explicitly considered in the supplementary material of Ishimoto *et al*.^35^. This may also be further understood from the fact the dominant feature of the model dynamics is sperm-sperm interaction, which is characterised by a cell separation which is much less than 15 *μ*m.

Hence the next step is to consider sperm-sperm interactions in detail – these are mediated by hydrodynamics and, possibly, steric effects, with the latter involving electrostatic repulsion and receptor-ligand adhesions^40^. As detailed in Supplementary Information, the mammalian flagellum diameter is much smaller than the variation in height of boundary accumulated sperm above a surface^39^. Hence, flagella are generally expected to pass over each other without steric influences, as further indicated by observations that flagella smoothly cross in the microscopy of multiple swimming sperm^41^. The prospective impact of steric interactions involving sperm heads is assessed in Supplementary Information. and shown to be negligible to an excellent approximation. Consequently, steric interactions are also neglected below and we focus on hydrodynamics.

With the number of cells denoted by *N*, we proceed by taking the equations of motion for the *n*th cell, with *n* ∈ {1, 2, …, *N*}, to be given by

1 $$\frac{d{{\boldsymbol{X}}}^{(n)}}{dt}={{\boldsymbol{U}}}^{(n)}:\,={{\boldsymbol{U}}}^{(n),{single}}+{{\boldsymbol{U}}}^{(n),others}$$

and

2 $$\frac{d{\theta }^{(n)}}{dt}={{\rm{\Omega }}}^{(n)}={{\rm{\Omega }}}^{(n),others}$$

for the two-dimensional positions ***X***^(*n*)^ and the angles *θ*^(*n*)^.

Thus, an isolated sperm is modelled as moving in a straight line at the experimentally obtained, and hence known a priori, swimming velocity ***U***^(*n*),*single*^, which is observed to very good approximation^9^. However, in the presence of other cells, the velocity of the cell is modified by ***U***^(1),*others*^ for cell 1 and analogously for the remaining cells. These modifier velocities are a priori unknown two-dimensional vectors and thus constitute a total of 2*N* scalar unknowns. In addition, the rate of rotation of cell 1 in the plane of swimming is modified by Ω^(1),*others*^, and similarly for the other cells. Hence, there is a total of 3*N* scalar unknowns a priori, namely

$$\{{{\boldsymbol{U}}}^{(1),others},\ldots ,{{\boldsymbol{U}}}^{(N),others}\},$$

and

$$\{{{\rm{\Omega }}}^{(1),others},\ldots ,{{\rm{\Omega }}}^{(N),others}\}.$$

These modifier velocities and angular velocities may be determined using the background flows resulting from the other cells that induce sperm-sperm interactions, as we proceed to discuss.

Let the triplet (*n*, *m*, *l*) refer to the *l*^*th*^ singularity in the approximation of the *m*^*th*^ PCA mode for the *n*^*th*^ cell, with ${{\boldsymbol{x}}}_{0}^{(n,m,l)},\,{\varepsilon }^{(n,m,l)}$ referring to location and regularization parameter. Then, exploiting the linearity of the Stokes equations governing the fluid dynamics, the flow generated at ${{\boldsymbol{x}}}_{0}^{(n,m,l)}$ due to the other cells, denoted ${u^{\prime} }_{t}({{\boldsymbol{x}}}_{0}^{(n,m,l)},t)$, is given by a superposition of the flows generated by the singularities associated with regularised Stokeslets representations of the other cells. Hence

3 $${u^{\prime} }_{i}({{\boldsymbol{x}}}_{0}^{(n,m,l)},t):\,=\sum _{n^{\prime} =1,n^{\prime} \ne n}^{N}\,\sum _{m^{\prime} =0}^{M}\,\sum _{l^{\prime} =1}^{L(m^{\prime} )}\,\sum _{j=1}^{3}\,{G}_{ij}{\,f}_{j}^{m^{\prime} ,n^{\prime} }$$

where ${G}_{ij}:\,={G}_{ij}({{\boldsymbol{x}}}_{0}^{(n,m,l)},{{\boldsymbol{x}}}_{0}^{(n^{\prime} ,m^{\prime} ,l^{\prime} )};{\varepsilon }^{(n^{\prime} ,m^{\prime} ,l^{\prime} )})$ and ${f}_{j}^{m^{\prime} ,n^{\prime} }:\,={f}_{j}^{(m^{\prime} )}({\varphi }^{(n^{\prime} )}(t)).$ With the *n*^*th*^ cell centroid defined by

$${{\boldsymbol{X}}}_{c}^{(n)}:\,=\frac{1}{K}\,\sum _{m=0}^{M}\,\sum _{l=1}^{L(m)}\,{{\boldsymbol{x}}}_{0}^{(n,m,l)},$$

where $K={\sum }_{m=0}^{M}\,L(m),$ we further define the residual velocity

$${{\boldsymbol{u}}}_{res}^{(n,m,l)}:\,={\boldsymbol{u}}^{\prime} ({{\boldsymbol{x}}}_{0}^{(n,m,l)},t)-{{\boldsymbol{U}}}^{(n),others}-{{\rm{\Omega }}}^{(n),others}{{\boldsymbol{e}}}_{z}\times {{\boldsymbol{x}}}_{rel}^{(n,m,l)},$$

with ***e***_*z*_ the unit vector into the fluid perpendicular to the surface, and the relative location defined by

4 $${{\boldsymbol{x}}}_{rel}^{(n,m,l)}:\,={{\boldsymbol{x}}}_{0}^{(n,m,l)}-{{\boldsymbol{X}}}_{c}^{(n)}.$$

Then ***U***^(*i*),*others*^, Ω^(*i*),*others*^ for *i* ∈ {1, …, *N*} are determined by the *N* velocity closure equations

5 $${\bf{0}}=\sum _{m=0}^{M}\,\sum _{l=1}^{L(m)}\,{{\boldsymbol{u}}}_{res}^{(n,m,l)},\,n\in \{1,\ldots ,N\},$$

and the *N* angular velocity closure equations

6 $${\bf{0}}=\sum _{m=0}^{M}\,\sum _{l=1}^{L(m)}\,{{\boldsymbol{x}}}_{rel}^{(n,m,l)}\times {{\boldsymbol{u}}}_{res}^{(n,m,l)},\,n\in \{1,\ldots ,N\},$$

which are motivated and detailed in the Methods section.

Note that equation (5) constitutes 2*N* scalar equations as the velocities are two-dimensional vectors, whereas equation (6) is *N* scalar equations since the cross product of the two vectors, both of which lie in the swimming plane, is perpendicular to the swimming plane. Hence, the closure equations give 3*N* scalar constraints for the above 3*N* a priori unknowns; the latter can thus be determined given the location, velocity and angular velocity of each sperm, as explicitly demonstrated in the Methods section. Furthermore, the location, velocity and angular velocity of each sperm at initial time is known from the initial conditions and hence the modifier velocities and the modifier angular velocities can be determined at the initial time, allowing a numerical timestep of equations (1) and (2). Iterating thus generates the dynamics of the population, incorporating the information contained within the regularised singularity representation of each sperm. Further details, such as the specification of the initial conditions, the numerical timestepping scheme and how to solve the closure conditions are given in the Methods section.

Hereafter, we non-dimensionalise the system, setting the flagellar length, the beat period and the fluid viscosity to unity for both the LVM and HVM cases. The sperm collective dynamics has subsequently been simulated, using the above equations and assuming the cells swim in a doubly-periodic square box of length *L* = 5. Note that, sperm clustering is a relatively local phenomenon^4^ and we have illustrated that once *L* = 5 or larger, simulation predictions are insensitive to further increases in domain size, as detailed in the Supplementary Information.

### Collective dynamics with temporal fluctuations

**Figure 3:**
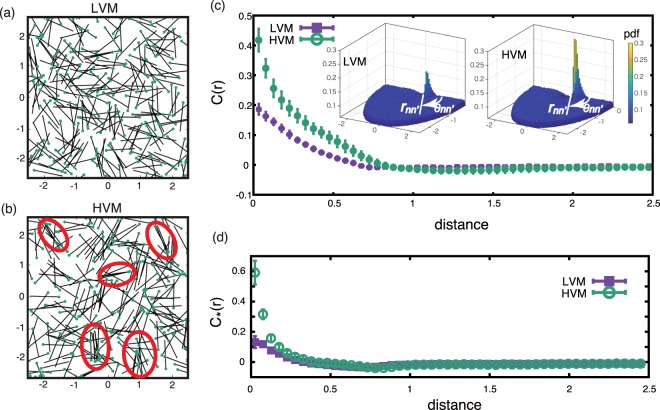
Snapshots of cell configurations for the simulation of *N* = 250 sperm cells with a domain length of *L* = 5, no averaging and the temporally varying velocity profile associated with the mean and the first *M* = 2 flow PCA modes. (**a**) The LVM case. The dots and the rods illustrate the position of each cell head and the associated cell orientation. (**b**) The HVM case, analogous to (**a**), with clustering cells highlighted by red circles. (Also see SM Movies). (**c**) The alignment ordering function, *C*(*r*), and (**d**) the clustering order function, ${C}_{\ast }(r)$, for the same simulations as (**a**,**b**), with bars denoting the standard error. ((**c**), inset) The predicted probability density functions for these simulations; the horizontal plane of the plots consists of the polar coordinates, *r*_*nn*′_, *θ*_*nn*′_.

We first consider a temporally varying velocity profile around a sperm, with no averaging of the trajectories over the flagellum beat period, conducting simulations with the velocity profile associated with the mean and *M* = 2 flow PCA modes. In Fig. 3(a,b), snapshots of the sperm configurations are shown. For the LVM case, the sperm cells regularly cross each other and are relatively uniform in their spatial distribution, whereas clustering can be observed in HVM, as highlighted by the red circles of Fig. 3b. Movies are available in the Supplementary Information and clearly show that the sperm clusters are transient, with the individual sperm in a given cluster changing over time, and also with clusters repeatedly emerging and disappearing.

To proceed, let the distance and angle between cells *n* and *n*′ be denoted by ${r}_{nn^{\prime} }:\,=|{{\boldsymbol{X}}}^{(n)}-{{\boldsymbol{X}}}^{(n^{\prime} )}|$ and let ${\theta }_{nn^{\prime} }:\,={\theta }^{(n)}-{\theta }^{(n^{\prime} )}$. The frequency histogram for (*r*_*nn*′_, *θ*_*nn*′_) is used to generate *p*(*r*_*nn*′_, *θ*_*nn*′_), the probability distribution function, which is plotted in the inset of Fig. 3(c) and exhibits significant peaks at *r*_*nn*′_ ≈ 0 and *θ*_*nn*′_ ≈ 0. The relative suppression of the histogram peak in LVM contrasts the HVM case, where the localization for small *r*_*nn*′_ highlights clustering as the cells are close to each other, while the localization for small *θ*_*nn*′_ shows alignment, as cells are essentially pointing in the same direction. This observation of simultaneous clustering and alignment has also been previously reported^11,16^.

We further characterise cell alignment, cell clustering and angular auto-correlation, with technical details concerning the plotting of these features reserved for the Methods section below. In particular, for cell alignment we define *C* = 〈cos *θ*_*nn*′_〉_*nn*′_, where the bracket ${\langle \cdot \rangle }_{nn^{\prime} }$ denotes both pairwise and temporal averaging. This ordering function depends on the relative cell separation, *r*, and is plotted in Fig. 3(c), with *N* = 250 sperm, for both LVM and HVM cases, with the bars denoting the standard error. These plots demonstrate substantially more alignment between two cells that are close in HVM. We similarly consider the cluster ordering function given by ${C}_{\ast }(r):\,=q(r)/{q}_{c}-1$, where $q(r):\,={\int }_{0}^{2\pi }\,{\rm{d}}\theta p(r,\theta )$, with *q*_*c*_ given by replacing the predicted probability density function, *p*, plotted in Fig. 3(c, inset) with the constant one for the same geometry and cell numbers. Hence ${C}_{\ast }(r)$ is high when there are more cells per unit area and Fig. 3(d) demonstrates substantially more close range clustering in HVM.

**Figure 4:**
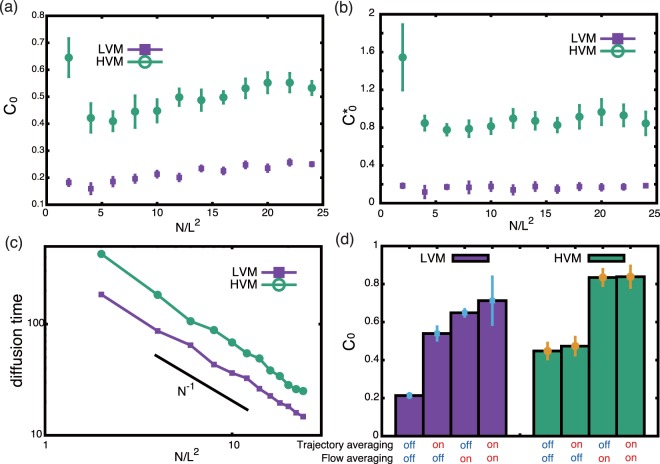
With a domain length of *L* = 5, the cell-number dependence for (**a**) the alignment order parameter *C*_0_, (**b**) the clustering order parameter ${C}_{0}^{\ast }$ and (**c**) the angular diffusion timescale. In these plots we have the end time, *T*_*end*_, is given by 6000 for *N*/*L*^2^ = 2, by 4000 for *N*/*L*^2^ = 4, by 2000 for *N*/*L*^2^ ∈ $[6,14]$, by 1000 for *N*/*L*^2^ ∈ $[16,20]$ and by 600 for *N*/*L*^2^ ∈ $[22,24]$. (**d**) The order parameter, *C*_0_, with all combinations of flow and trajectory averaging with *L* = 5, *N* = 250 cells and *T*_*end*_ = 2000. The bars in plots (**a**,**b**,**d**) correspond to the 95% fitting confidence interval, as detailed in the Methods section.

Furthermore, both clustering and alignment measures are higher in HVM across different cell densities, as summarised by Fig. 4(a,b) where, respectively, the alignment and clustering order parameters, ${C}_{0}:\,=C(0)$ and ${C}_{0}^{\ast }:\,={C}_{\ast }(0)$, are plotted. We proceed to consider angular auto-correlation via 〈cos (*θ*_*nn*′_(*t*) − *θ*_*nn*′_(*t* − *T*))〉_*nn*′_, which can be fitted to the exponential exp(−*T*/*τ*), with the parameter *τ* defining the angular diffusive timescale of correlation decay. A plot of *τ* in Fig. 4c highlights inversely proportionality to the cell density, suggesting angular diffusion arises mainly from pairwise interactions^16^.

### The impact of temporal averaging

Given the observed simultaneous clustering and alignment, both features can be further quantified by one of the ordering functions and we consider *C*(*r*), the alignment function, below. In particular, we proceed to classify the collective motion of the cells with the flow profile and trajectory averaged over the flagellum beat period, *i*.*e*. *M* = 0 (flow averaging), and ***U***^*single*^ = 〈***U***^*single*^〉_*traj*_ (trajectory averaging), with the bracket denoting time-averaging over the flagellum beat period. Such simplifying averaging is frequently considered^11,16^ and thus its impact is explored.

Firstly, note that yawing in the LVM case is subdued on temporally averaging the cell trajectories over the beat cycle, while the LVM oscillation between puller and pusher dynamics is subdued by averaging the fluid velocity over the flagellum beat cycle. Hence we consider the alignment order parameter and clustering surrogate, *C*_0_, with all combinations of whether the sperm fluid flow is averaged over the flagellum beat cycle or not, whether the sperm trajectory is averaged over the beat cycle or not and the choice of LVM or HVM; see Fig. 4(d). With full temporal resolution this highlights extensively more clustering in HVM (*C*_0_ = 0.45) compared to LVM (*C*_0_ = 0.21), a 114% increase. One can also observe that trajectory averaging essentially has no effect in HVM, with *C*_0_ = 0.47 after this averaging, as expected since there is minimal yawing in HVM.

To consider the impact of yaw, we thus review the LVM case, where applying trajectory averaging in the absence of flow averaging – which removes yaw – induces significantly more clustering, with *C*_0_ increasing from 0.21 to 0.54. Flow averaging also enhances the clustering surrogate *C*_0_ from 0.21 to 0.65. Hence both the cell yaw and the flows associated with the transient, fast timescale, pulling swimmer dynamics inhibit the clustering for sperm swimming within LVM.

With full temporal averaging, as often used in past studies^11,16^, one has *C*_0_ = 0.71 for LVM and *C*_0_ = 0.84 for LVM. This is a subtle, 18%, difference and even the LVM dynamics exhibits much more clustering than HVM collective behaviour in the absence of temporal averaging. Thus, with temporal averaging extensive alignment and clustering is always predicted, with much less difference between LVM and HVM collective behaviours, compared to modelling predictions with full temporal resolution, while only the results of the latter are reflected in observation^4^. This not only emphasises the significant impact of the finer-scale temporal dynamics on the populations, but also the need to incorporate such resolution within coarse-grained approaches.

## Discussion

Motivated by observations from bovine studies^4^ we have numerically investigated sperm clustering, modelling sperm as superpositions of flow singularities, coarse-grained from experimentally obtained human sperm digital microscopy, in both a low viscosity medium and a highly viscous–weakly elastic medium by the application of principal component analysis. In particular, this has provided coarse-grained data accommodating highly resolved spatio-temporal information for studying collective behaviour, which also allows us to consider whether temporal averaging over the flagellum beat cycle is an accurate simplification.

Firstly, including fine-scale temporal dynamics, one finds simulations for LVM predict extensively less clustering than for HVM, with the latter simulations exhibiting a dynamic clustering, as reflected in Tung *et al*.’s study^4^. However, one must be cautious about relating our predictions to these experimental studies given the difference in sperm species and that the polyacrylamide medium in their study is beyond our assumption of linear viscoelasticity. Hence, detailed comparison is however outside our scope, though we note that nonlinear viscoelasticity could further enhance the clustering^11^.

Nonetheless, a fundamental and general feature of our results arises on analysing the impact of averaging over the velocity field and the sperm-swimming trajectory. In particular, this emphasises that the presence of both cell yaw and swimmer pulling due to the fast dynamics are the core reasons why the virtual sperm cells do not dynamically cluster more extensively in the LVM case. Furthermore, the results emphasise in general that the detailed flagellar waveform is important in determining differences in sperm behaviour within different rheological media^42^ for the population dynamics as well as at the level of an individual cell.

A further result is that the temporal averaging of either the flow or the trajectories induces an overestimation of clustering. This in turn generically demonstrates that coarse-grained studies with a temporal averaging over the fine details of the flagellum beat period are too coarse and can be misleading. Hence one cannot neglect the complication that the fast timescale details of the flagellum beat significantly influence larger scale sperm behaviour, whether in modelling or considering the impact of cell level observations on population dynamics. More generally, such observations not only justify developing numerically efficient coarse-graining methods that incorporate fast dynamics, but also motivate the need for further high-speed microscopy of the sperm flagellum, together with analogous studies for other microswimmers.

## Methods

### Images and flow

We have used the human digital video microscopy of reference^9^, which imaged sperm that had penetrated approximately 2 cm into a capillary tube and were in the region of cell boundary accumulation, about 10–20 *μ*m from the capillary tube surface. Two cases were considered: (i) sperm motility in a watery *in*-*vitro* fertilization medium and (ii) Earle’s balanced salt solution with the addition of 1% methylcellulose. We treat the latter medium as a linear Maxwell fluid since it exhibits storage and loss moduli consistent with a Maxwell fluid possessing an elastic relaxation time of *τ* = 0.006 s, an effective viscosity of 0.14 Pa · s and a weakly viscoelastic Deborah number of De = 0.2^9^. Throughout, we distinguish these cases via the label of low-viscosity medium (LVM) and high-viscosity medium (HVM) for brevity. The digitised flagellum images have previously been reconstructed as a limit cycle trajectory in a low-dimensional PCA phase space to give a simple but accurate representation of the flagellar waveform^35,36^. Applying this PCA waveform in the two types of medium as a boundary condition of the inertialess fluid equations, the flow field around the human sperm has previously been computed via the boundary element method, using the Newtonian Stokes equations for LVM and the linear Maxwell equations for HVM (Fig. 1(b,c) ^35–37^), as we summarise below.

### Microswimming in a linear Maxwell fluid

Given the fluid flows induced by sperm swimming possess only negligible Reynolds number we have by momentum balance that $\nabla \cdot {\boldsymbol{\sigma }}={\bf{0}}$, where ***σ*** is the stress tensor. These equations are coupled with the constraint of fluid incompressibility, that is $\nabla \cdot {\boldsymbol{u}}=0$ for the velocity field ***u***, as well as no-slip conditions on the sperm and zero flow boundary conditions at spatial infinity, so that

$${\boldsymbol{u}}({\boldsymbol{x}},t)={{\boldsymbol{u}}}_{S}({\boldsymbol{x}},t),\,{\boldsymbol{x}}\in S,\,{\boldsymbol{u}}\to 0\,{\rm{as}}\,|{\boldsymbol{x}}|\to \infty ,$$

where ***u***_*S*_(***x***, *t*) is the velocity on the surface of the sperm cell, *S*. In particular, ***u***_*S*_(***x***, *t*) can be written in terms of the flagellar beat pattern, as extracted from video microscopy and principal component analysis, together with the velocity ***U*** and angular velocity **Ω** of the cell fixed reference frame. These latter two vectors are not imposed a priori, but simultaneously determined with the calculation of the velocity vector field.

Such calculations also require the specification of a constitutive relation. For a Newtonian fluid this is

$${\sigma }_{ij}=-\,p{\delta }_{ij}+{\tau }_{ij},\,i,j\in \{1,2,3\},$$

where *p* denotes the pressure field *p* and ***τ*** denotes the deviatoric stress, and is defined by

$${\boldsymbol{\tau }}=2\mu {\boldsymbol{D}},\,{\rm{where}}\,{D}_{ij}=\frac{1}{2}\,(\frac{\partial {u}_{i}}{\partial {x}_{j}}+\frac{\partial {u}_{j}}{\partial {x}_{i}})$$

with *μ* denoting the viscosity of the fluid. For a linear Maxwell fluid the constitutive relation is altered in terms of the deviatoric stress, which is instead given by

$$\lambda \frac{\partial {\boldsymbol{\tau }}}{\partial t}+{\boldsymbol{\tau }}=2\mu {\boldsymbol{D}},$$

for an elastic relaxation time, *λ*. In this case, an explicit time derivative entails that an initial condition for the velocity field must also be specified.

Sufficiently far in the past, at initial time *t*_0_, no flow and constant pressure are assumed with

$${\boldsymbol{U}}({t}_{0})={\boldsymbol{\Omega }}({t}_{0})={\bf{0}}$$

and the flagellar waveform is taken to be of zero velocity, and evolved to the observed waveform on a short timescale, though not so short that inertia is important. Since the memory of the initial conditions decays on a timescale of *λ*, we have that once $t-{t}_{0}\gg \lambda$ these initial conditions have negligible effect on the solution and thus they may be used without loss of generality.

Below we summarise why the velocity flow field and sperm trajectory are predicted to be the same for both a linear Maxwell fluid and a Newtonian fluid given the flagellar beat pattern of the cell. Hence Newtonian boundary element methods can be used to determine the flow fields for sperm swimming in the high viscosity-weakly elastic media considered in this paper.

First, the Newtonian momentum equations are given by

$$-\frac{\partial p}{\partial {x}_{i}}+\mu {\nabla }^{2}{u}_{i}=0.$$

In contrast, one has

$$-\frac{\partial }{\partial {x}_{i}} {\mathcal L} \,{p}^{M}+\mu {\nabla }^{2}{u}_{i}^{M}=0,$$

for the linear Maxwell fluid, with *p*^*M*^ and ***u***^*M*^ denoting the Maxwell fluid pressure and velocity flow field, obtained by applying the operator ${\mathcal L} :\,=(1+\lambda \partial /\partial t)$ to the momentum balance equations. Hence

$${u}_{i}^{M}={u}_{i},\,{p}^{M}={ {\mathcal L} }^{-1}p,$$

are solutions and satisfy all boundary conditions. For the initial time *t*_0_ the flow and sperm are stationary and hence the linear Maxwell solution and Newtonian solution coincide and thus the initial conditions also hold. These initial conditions also entail that the inverse operator ${ {\mathcal L} }^{-1}$ is unique as required for a unique pressure field for the linear Maxwell fluid solution. Hence, by explicit construction, the linear Maxwell and Newtonian bulk velocity fields are identical. Furthermore, since the bulk velocity fields are identical, so are the surface velocity fields by continuity and hence so are ***U*** and **Ω**, the remaining elements of the solution. However, the pressure, stress, viscous drag and other aspects of the mechanical forces required to maintain the sperm movement differ for the linear Maxwell fluid compared to Newtonian media.

A more formal proof of the above result, together with details of the boundary element calculations of the cell trajectories and velocity flow fields in linear viscolelastic media, can be found in recent work^37^. In addition, details of Newtonian flow calculations may be found in numerous older works, for example^43,44^.

### Closure equations

The closure equations 5 and 6 can be interpreted via a representation of the sperm as an extended object, consisting of *K* negligibly small spheres, where $K={\sum }_{m=0}^{M}\,L(m)$ is the number of regularised Stokeslets in a single sperm. This is motivated by the commonly used simplification of sphere-linked swimmer models^45–47^, noting that the regularised Stokeslets are approximately singular Stokeslets, at least away from the singularity, and similarly Stokeslets are good approximations for spheres away from the very near field of the sphere.

To proceed, for the Newtonian case, we take the location of each sphere to be given by that of the regularised Stokeslets in Fig. 2. Let *c* be the radius of each point-like spherical particle, and *μ* the viscosity of the medium. Then using the Stokes drag law gives the hydrodynamic force on the sphere centred at ${{\boldsymbol{x}}}_{0}^{(n,m,l)}$

7 $${{\boldsymbol{F}}}^{(n,m,l)}=\xi ({{\boldsymbol{u}}}_{res}^{(n,m,l)}-{{\boldsymbol{U}}}^{(n),{single}})+{{\boldsymbol{f}}}^{(m,n)},$$

where *ξ* = 6*πμc* is a constant and the residual velocity ${{\boldsymbol{u}}}_{res}^{(n,m,l)}$ is defined in the main text. In particular the velocity term ${{\boldsymbol{u}}}_{res}^{(n,m,l)}-{{\boldsymbol{U}}}^{(n),single}$ constitutes the speed of the sphere relative to the background flow, and the force term ***f***^(*m*,*n*)^ is the force due to the singularity. The total force balance on the *n*^*th*^ sperm is then given by

8 $$\sum _{m=0}^{M}\,\sum _{l=1}^{L(m)}\,{{\boldsymbol{F}}}^{(n,m,l)}={\bf{0}}.$$

We first consider the case of one sperm, labelled by (*n*), swimming in isolation. Then ${{\boldsymbol{u}}}_{res}^{(n,m,l)}={\bf{0}}$ by the definition of this residual velocity and we have

9 $$-\xi K{{\boldsymbol{U}}}^{(n),{single}}+\sum _{m=0}^{M}\,\sum _{l=1}^{L(m)}\,{{\boldsymbol{f}}}^{(n,m)}={\bf{0}},$$

where $K={\sum }_{m=0}^{M}\,L(m)$. As all terms in the above are independent of the other cells, this still holds even if other cells are present. Hence, combining Equations (7), (8) and (9) the force balance on each cell collapses to

10 $$\xi \,\sum _{m=0}^{M}\,\sum _{l=1}^{L(m)}\,{{\boldsymbol{u}}}_{res}^{(n,m,l)}={\bf{0}},\,n\in \{1,\ldots ,N\},$$

yielding the first closure equation (5).

Similarly, the moment balance relation for the *n*^*th*^ sperm around its centroid is given by

11 $$\sum _{m=0}^{M}\,\sum _{l=1}^{L(m)}\,{{\boldsymbol{x}}}_{rel}^{(n,m,l)}\times {{\boldsymbol{F}}}^{(n,m,l)}={\bf{0}},$$

where the relative location ${{\boldsymbol{x}}}_{rel}^{(n,m,l)}$ is given in the main text. This generates the second closure equation,

12 $${\bf{0}}=\sum _{m=0}^{M}\,\sum _{l=1}^{L(m)}\,{{\boldsymbol{x}}}_{rel}^{(n,m,l)}\times {{\boldsymbol{u}}}_{res}^{(n,m,l)},\,n\in \{1,\ldots ,N\},$$

on again noting there is no net moment associated with a single cell, swimming in isolation. Solving these closure equations with respect to the ***U***^(*n*),*others*^ and Ω^(*n*),*others*^, we have explicit forms,

13 $${{\boldsymbol{U}}}^{(n),others}=\frac{1}{K}\,\sum _{m=0}^{M}\,\sum _{l=1}^{L(m)}\,{\boldsymbol{u}}^{\prime} ({{\boldsymbol{x}}}_{0}^{(n,m,l)})$$

and

14 $${{\rm{\Omega }}}^{(n),others}{{\boldsymbol{e}}}_{z}=\frac{{\sum }_{m=0}^{M}\,{\sum }_{l=1}^{L(m)}\,{{\boldsymbol{x}}}_{rel}^{(n,m,l)}\times {\boldsymbol{u}}^{\prime} ({{\boldsymbol{x}}}_{0}^{(n,m,l)})}{{\sum }_{m=0}^{M}\,{\sum }_{l=1}^{L(m)}\,|{{\boldsymbol{x}}}_{rel}^{(n,m,l)}{|}^{2}}.$$

In the case of the linear Maxwell fluid, equations (7), (8), (9) and (11) hold on making the substitutions

$${{\boldsymbol{F}}}^{(n,m,l)}\to { {\mathcal L} }^{-1}{{\boldsymbol{F}}}^{(n,m,l)},\,{{\boldsymbol{f}}}^{(m,n)}\to { {\mathcal L} }^{-1}{{\boldsymbol{f}}}^{(m,n)},$$

where ${\mathcal L} =(1+\lambda \partial /\partial t)$. In particular, the cancellations required to derive equations (10) and (12), together in turn with equations (13) and (14), proceed identically to the Newtonian case, providing identical closure equations for the linear Maxwell fluid.

#### Numerical implementation

A solution is required for the ordinary differential equations governing the population dynamics, (1) and (2), subject to initial conditions and the closure equations, which reduce to the explicit relations of equations (13) and (14) for the modifier velocity, ***U***^(*n*),*others*^, and the modifier angular velocity, Ω^(*n*),*others*^***e***_*z*_.

Noting the location, velocity and angular velocity of each sperm is known from initial conditions the modifier velocities and angular velocities can be determined at the initial time from equations (13) and (14). In turn this allows a numerical timestep of equations (1) and (2); iterating thus generates the dynamics of the population, while incorporating the information contained within the regularised singularity representation of each sperm. In practice, the time evolution is performed simply, by the explicit Euler method, with the time discretization Δ*t* = *T*/100, where *T* = 1 is the non-dimensional time period of the flagellar beat cycle.

To specify the initial conditions in the simulations, sperm positions and orientations are drawn from a uniform random distribution on the square domain. Note that the sperm configuration rapidly relaxes to its statistical stationary state regime, regardless of the initial conditions, and that the simulation with initial complete alignment exhibits an analogous rapid convergence. The simulations are performed from *t* = 0 to *t* = *T*_*end*_, where the end time, *T*_*end*_, ranges from 1000 to 6000, depending on the number of cells, *N*. This end-time is always much larger than time required for the system to relax to the statistical steady state and once this constraint is satisfied the results have been confirmed to be insensitive to the choice of *T*_*end*_. The computations have been performed within the supercomputing system at the Institute for Information Management and Communication (IIMC), Kyoto University. Using 36 cores in parallel computation required approximately 400 CPU hours to simulate *N* = 250 sperm up to *t* = *T*_*end*_ = 2000.

#### Plotting features of the population data analysis

As documented in the main text, the relative distance, *r*_*nn*′_, and the relative angle, *θ*_*nn*′_, between the cells *n* and *n*′ are extensively analysed via cell alignment, clustering and angular auto-correlation. The alignment ordering function, *C* = 〈cos *θ*_*nn*′_〉_*nn*′_ is a function of the cell separation, denoted *r*. Dividing the time interval [0, *T*_*end*_] into 10 sub-intervals and averaging both pair-wise and temporally within each of these time sub-intervals, leads to the plots of *C*(*r*) as in Fig. 3(c) of the main text for both HVM and LVM cases. Here, the bars give the standard error, that is plus/minus the standard deviation for the calculations from these 10 sub-intervals.

The analogous clustering order function ${C}_{\ast }(r)$, together with standard errors, is also calculated via the mean and standard deviation across the above-mentioned 10 time sub-intervals and plotted in Fig. 3(d) of the Main Text. The fact ${C}_{\ast }(r)$ can be negative is expected from its definition; for example when more clustering is observed at short range, small, *r* then conservation of cell number entails that sperm must be more dispersed than the homogeneous distribution elsewhere, leading to negative ${C}_{\ast }(r)$ for larger values of *r*.

Both these ordering functions have global maxima as *r* → 0^+^, and this is a useful summary statistic. Hence we plot the alignment order parameter *C*_0_ = *C*(0), and the clustering order parameter ${C}_{0}^{\ast }={C}_{\ast }(0)$ in Fig. 4(a,b) of the main text. However, it not feasible to numerically take *r* → 0^+^ as this ultimately reduces *r* below numerical resolution. Thus these plots are generated by an exponential fitting for both *C*(*r*) and ${C}_{\ast }(r)$ using the MATLAB fit function, allowing *C*_0_ and ${C}_{0}^{\ast }$ to be determined, together with 95% confidence intervals via the MATLAB function confit, with the latter plotted as bars in Fig. 4. Finally, the auto-correlation function 〈cos (*θ*_*nn*′_(*t*) − *θ*_*nn*′_(*t* − *T*))〉_*nn*′_, is fitted by exp(−*T*/*τ*), with *τ* presented in Fig. 4. However this plot is not accompanied by confidence intervals as the latter are too small to be meaningfully depicted.

## Electronic supplementary material

Supplementary Movie 1

Supplementary Movie 2

Supplemental Materials

## Data Availability

Custom computer codes simulating the collective behaviour are available to readers via the University of Oxford Research Archive: https://doi.org/10.5287/bodleian:ZBRnm2Pa9.

## References

[1] Zinaman M, Brown C, Selevan S, Clegg E (2000). Semen quality and human fertility: A prospective study with healthy couples. J. Androl..

[2] Rothschild L (1949). Measurement of sperm activity before artificial insemination. Nature.

[3] Suarez SS, Pacey AA (2006). Sperm transport in the female reproductive tract. Hum. Reprod. Update.

[4] Tung CK (2017). Fluid viscoelasticity promotes collective swimming of sperm. Sci. Rep..

[5] Moore H, Dvorakova K, Jenkins N, Breed W (2002). Exceptional sperm cooperation in the wood mouse. Nature.

[6] Joanny MCMJF (2013). Hydrodynamics of soft active matter. Rev. Mod. Phys..

[7] Elgeti J, Winkler RG, Gompper G (2015). Physics of microswimmers- single particle motion and collective behevior: a review. Rep. Prog. Phys..

[8] Suarez SS, Dai X (1992). Hyperactivation enhances mouse sperm capacity for penetrating viscoelastic media. Biol. Reprod..

[9] Smith DJ, Gaffney EA, Gadêlha H, Kapur N, Kirkman-Brown J (2009). Bend propagation in the flagella of migrating human sperm, and its modulation by viscosity. Cell Motil. Cytoskel.

[10] Nosrati R, Driouchi A, Yip CM, Sinton D (2015). Two-dimensional slither swimming of sperm within a micrometre of a surface. Nat. Commun..

[11] Li G, Ardekani AM (2016). Collective motion of microorganisms in a viscoelastic fluid. Phys. Rev. Lett..

[12] Smith DJ, Gaffney EA, Blake JR, Kirkman-Brown JC (2009). Human sperm accumulation near surfaces: a simulation study. J. Fluid Mech..

[13] Friedrich B, Riedel-Kruse I, Howard J, Jülicher F (2010). High-precision tracking of sperm swimming fine structure provides strong test of resistive force theory. J. Exp. Biol..

[14] Elgeti J, Kaupp U, Gompper G (2010). Hydrodynamics of sperm cells near surfaces. Biophys. J.

[15] Schoeller S, Keveany E (2018). From flagellar undulations to collective motion: predicting the dynamics of sperm suspensions. J. R. Soc. Interface.

[16] Saintillan D, Shelly MJ (2007). Orientational order and and instabilities in suspension of self-locomotive rods. Phys. Rev. Lett..

[17] Zöttle A, Stark H (2014). Hydrodynamic determines collective motion and phase behavior of active colloids in quasi-two-dimensional confinement. Phys. Rev. Lett..

[18] Oyama N, Molina JJ, Yamamoto R (2016). Purely hydrodynamic origin for swarming of swimming particles. Phys. Rev. E.

[19] Sokolov A, Aranson IS (2012). Physical properties of collective motion in suspension of bacteria. Phys. Rev. Lett..

[20] Saintillan, D. & Shelley, M. Instabilities and pattern formation in active particle suspensions: Kinetic theory and continuum simulations. *Phys*. *Rev*. *Lett*. **100**, 178103 (2008). 10.1103/PhysRevLett.100.178103 18518342

[21] Ezhilan Barath, Shelley Michael J., Saintillan David (2013). Instabilities and nonlinear dynamics of concentrated active suspensions. Physics of Fluids.

[22] Peshkov, A., Aranson, I., Bertin, E., Chate, H. & Ginelli, F. Nonlinear field equations for aligning self-propelled rods. *Phys*. *Rev*. *Lett*. **109**, 268701 (2012). 10.1103/PhysRevLett.109.268701 23368625

[23] Dunkel J (2013). Fluid dynamics of bacterial turbulence. Phys. Rev. Lett..

[24] Brumley DR, Wan KY, Polin M, Goldstein RE (2014). Flagellar synchronization through direct hydrodynamic interactions. eLife.

[25] Fauci LJ (1990). Interaction of oscillating filaments: A computational study. J. Comput. Phys..

[26] Yang, Y., Elgeti, J. & Gompper, G. Cooperation of sperm in two dimensions: Synchronization, attraction, and aggregation through hydrodynamic interactions. *Phys*. *Rev*. *E* **78**, 061903 (2008). 10.1103/PhysRevE.78.061903 19256864

[27] Llopis I, Pagonabarraga I, Lagomarsino MC, Lowe CP (2013). Cooperative motion of intrinsic and actuated semiflexible swimmers. Phys. Rev. E.

[28] Olson SD, Fauci LJ (2015). Hydrodynamic interactions of sheets vs filaments: Synchronization, attraction, and alignment. Phys. Fluids.

[29] Simons J, Fauci L, Cortez R (2015). A fully three-dimensional model of the interaction of driven elastic filaments in a stokes flow with applications to sperm motility. J. Biomech..

[30] Yang, Y., Marceau, V. & Gompper, G. Swarm behavior of self-propelled rods and swimming flagella. *Phys*. *Rev*. *E* **82**, 031904 (2010). 10.1103/PhysRevE.82.031904 21230105

[31] Lindemann CB (1994). A model of flagellar and ciliary functioning which uses the forces transverse to the axoneme as the regulator of dynein activation. Cell Motil. Cytoskel..

[32] Lindemann CB (1994). A geometric clutch hypothesis to explain oscillations of the axoneme of cilia and flagella. J. Theor. Biol..

[33] Hilfinger A, Chattopadhyay AK, Julicher F (2009). Nonlinear dynamics of cilia and flagella. Phys. Rev. E.

[34] Oriola David, Gadêlha Hermes, Casademunt Jaume (2017). Nonlinear amplitude dynamics in flagellar beating. Royal Society Open Science.

[35] Ishimoto K, Gadelha H, Gaffney EA, Smith DJ, Kirkman-Brown J (2017). Coarse-graining the fluid flow around a human sperm. Phys. Rev. Lett..

[36] Ishimoto K, Gadelha H, Gaffney EA, Smith DJ, Kirkman-Brown J (2018). Human sperm swimming in a mucus analogue medium. J. Theor. Biol..

[37] Ishimoto K, Gaffney EA (2017). Boundary element methods for particles and microswimmers in a linear viscoelastic fluid. J. Fluid Mech..

[38] Cortez R (2001). The method of regularized stokeslets. SIAM J. Sci. Comput..

[39] Rothschild (1963). Non-random distribution of bull spermatozoa in a drop of sperm suspension. Nature.

[40] Ishimoto K, Gaffney EA (2016). Mechanical tuning of mammalian sperm behaviour by hyperactivation, rheology and substrate adhesion: a numerical exploration. J. R. Soc. Interface.

[41] Gadêlha H, Gaffney EA, Smith DJ, Kirkman-Brown JC (2010). Non-linear instability in flagellar dynamics: a novel modulation mechanism in sperm migration?. J. R. Soc. Interface.

[42] Kirkman-Brown JC, Smith DJ (2011). Sperm motility: is viscosity fundamental to progress?. Mol. Hum. Reprod..

[43] Pozrikidis, C. *A Practical Guide to Boundary Element Method with Software Library BEMLIB* (CRC Press, 2002).

[44] Ishimoto K, Gaffney EA (2014). A study of spermatozoan swimming stability near a surface. J. Theor.Biol..

[45] Najafi A, Golestanian R (2004). Simple swimmer at low Reynolds number: Three linked spheres. Phys. Rev. E.

[46] Alexander GP, Pooley CM, Yeomans JM (2008). Scattering of low-Reynolds-number swimmers. Phys. Rev. E.

[47] Vladimirov VA (2013). On the self-propulsion of an *N*-sphere micro-robot. J. Fluid Mech..

[48] Wolf DP, Blasco L, Khan MA, Litt M (1978). Human cervical-mucus. IV. Viscoelasticity and sperm penetrability during ovulatory menstrual-cycle. Fertil. steril..

