# Supplementary material for: Hydrodynamic Clustering of Human Sperm in Viscoelastic Fluids
Source: Sci Rep. 2018 Oct 22;8:15600. doi: 10.1038/s41598-018-33584-8 (PMC6197292; doi:10.1038/s41598-018-33584-8)
Supplement: Supplementary file 3 — Supplemental Materials [file 41598_2018_33584_MOESM3_ESM.pdf]

Supplementary Materials

Hydrodynamic Clustering of Human Sperm in Viscoelastic Fluids

Kenta Ishimoto[1,2] and Eamonn A. Gaffney[1]

[1]Wolfson Centre for Mathematical Biology, Mathematical Institute, University of Oxford, Oxford OX2 6GG, UK
[2]Graduate School of Mathematical Sciences, The University of Tokyo, Tokyo 153-8914, Japan

I. DOMAIN-SIZE DEPENDENCE

In the main text, we have analyzed the dynamics for a domain-size of $L = 5$, where lengthscales have been non-dimensionalised by the flagellum length. The sperm clustering dynamics is a relatively local phenomenon, and thus the domain-size does not affect the dynamical properties. In particular, we plot the alignment order parameter, $C_0 := C(0)$, the clustering order parameter $C_0^* := C_*(0)$ and the angular diffusion time, τ, in SM Figure 1 for simulations with the same cell density of N/L^2, but with different values of the domain-size, L. As illustrated in the figure, all results except for the smallest domain with $L = 2$ show similar values, demonstrating domain-size independence once the domain is of the size considered in the main text.

II. HEAD STERIC INTERACTIONS

In this supplementary section, the effects of steric interactions are briefly considered. As summarised in the main text, the mammalian flagellar diameter is $\approx$ 0.5-1μm, which is in turn smaller than the 15μm scale for the height variation of boundary accumulated sperm above a surface [6]. Hence, as mentioned in the main text, near a surface flagella are generally expected to pass over each other in virtually all cases without steric influences, as further indicated by the observations that flagella smoothly cross in high-resolution microscopy of multiple swimming sperm [2]. Thus steric interactions between sperm flagella are not considered and instead we focus on steric interactions involving the sperm head. Attractive head-head steric interactions would be relevant for rodent sperm train modelling, where sperm-sperm attachments form [5]. However, the fluctuating clustering of bovine sperm observed in Tung et al. [8], which does not involve attachment, instead motivates the consideration of repulsive steric forces.

Thus we proceed to consider the prospective impact of short-range repulsive interactions involving the sperm head in the current modelling framework of regularised Stokes flow singularities. We therefore introduce an additional velocity-field induced by the presence of steric influences associated with spherical sperm heads of radius a, in addition to the flow due to the other cells' hydrodynamic interaction with the surrounding media.

The additional velocity due to steric influences for the triplet (n, m, l) is modelled via

$$\boldsymbol{u}^{steric}(\boldsymbol{x}_0^{(n,m,l)}, t) = \sum_{n'=1, n'\neq n}^{N} b \frac{e^{-r_{n'}/a}}{1 - e^{-r_{n'}/a}} \frac{\boldsymbol{r}_{n'}}{|\boldsymbol{r}_{n'}|}. \tag{1}$$

Here the vector $\boldsymbol{r}_{n'}$ indicates the relative position of the point $\boldsymbol{x}_0^{(n,m,l)}$ from the centre of the sperm head associated with the n' cell, with the parameter b representing the strength of the short-range repulsion. The above expression is of the same form used for steric interactions in previous studies [3] and, in particular, once b is sufficiently large, any given cell head is an exclusion region for all other cells.

In SM Figure 2, we show the alignment ordering function, $C(r)$, and its associated order parameter $C_0 := C(0)$, obtained from the simulations with the repulsive strengths $b \in \{0, 1, 10, 100\}$ and the head-sizes $a \in \{0.03, 0.06\}$, noting the unit of length is a flagellum length. The human sperm head with dimensions 4.5μm$\times$2.8μm$\times$1.1μm possess the same volume as a sphere of radius $a \approx 0.032$ on using a flagellar length of 56μm to non-dimensionalize [7]. Bovine sperm have relatively larger heads, with $a \approx 0.05$ for *Bos taurus* [1]. The instantaneous non-dimensional velocity scale of the human sperm in low viscous medium is of order unity [4] and thus the range of steric interaction strength, which includes values with $b = 0$ and $b \gg 1$, covers cases with the absence of steric forces on one hand and extreme short-range repulsion on the other.

For each case of these steric interaction parameters, the alignment ordering functions almost coincide, as shown in the left and the middle columns of SM Figure 2, which indicates the head steric interactions do not impact the collective behavior. The right column of the figure also shows the alignment order parameter, C_0 is not affected. These results strongly support and motivate the assumption of negligible head steric interactions used in the main text.

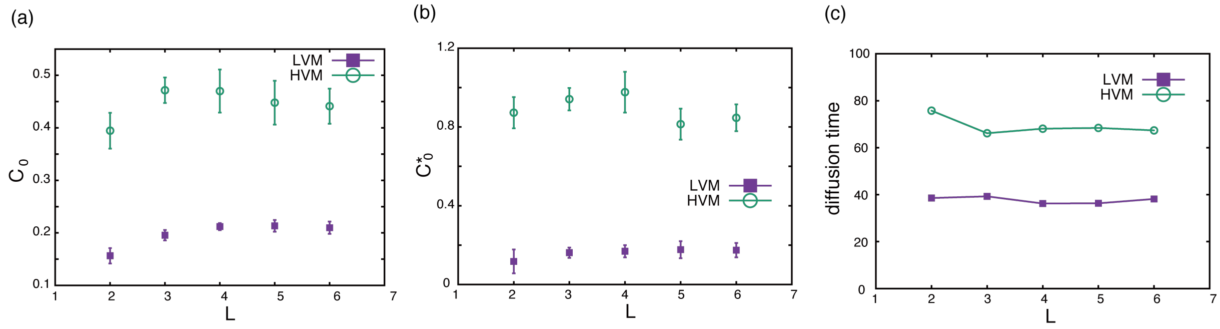

Figure 1. The dependence of statistical measures predicted by simulation for varying domain size, L in units of flagellum length, with a fixed cell density of $N/L^2 = 10$ in both the LVM and HVM cases, where N is the cell number. The end time, T_{end}, for this plot is given by $T_{end} = 6000$ for $N \in \{40, 90\}$, $T_{end} = 2000$ for $N \in \{160, 250\}$ and, finally, $T_{end} = 1000$ for $N = 360$. (a) The alignment order parameter, C_0. (b) The cluster order parameter, C_0^*. (c) The angular diffusion time, τ, as defined in the main text. Please also see the Methods section for details on the bars in plots (a), (b), which give the range corresponding to the 95% confidence intervals generated in calculating the order parameters.

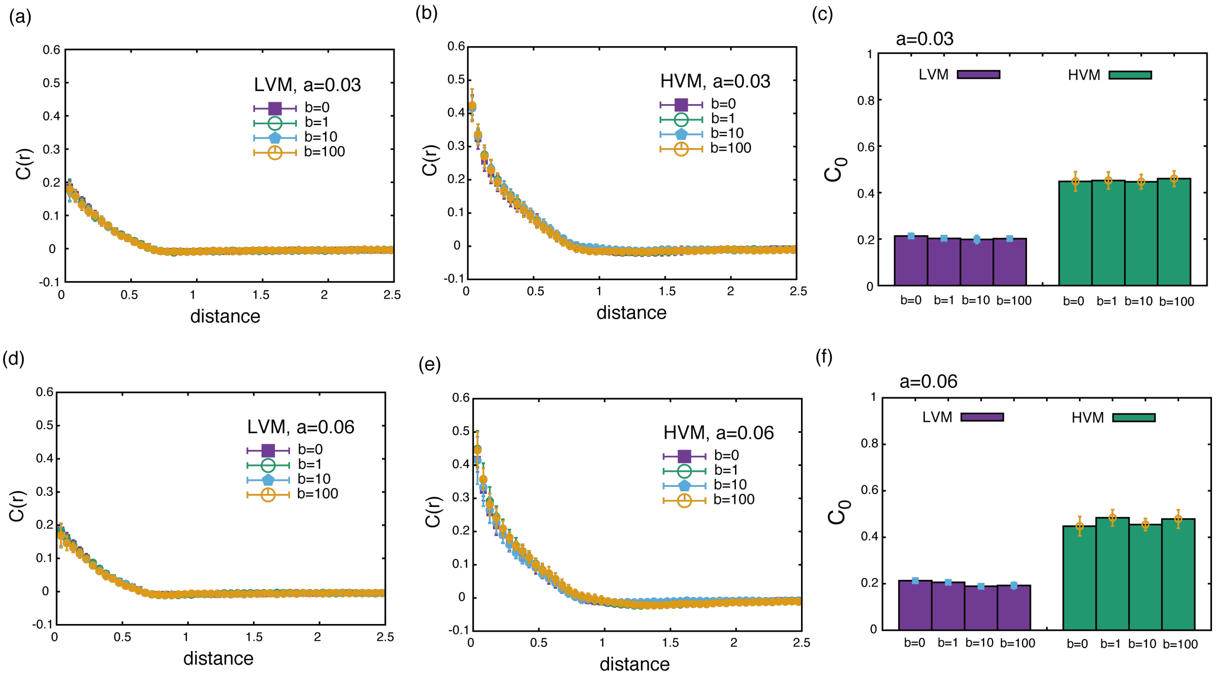

Figure 2. The alignment ordering functions, $C(r)$, and its associated order parameter C_0, when sperm head steric interactions are considered. In the upper panels (a)-(c), the head radius parameter is given by $a = 0.03$, and the results for a larger head case $a = 0.06$ are shown in the lower panels (d)-(f). The cell density and the domain-size are, respectively, $N/L^2 = 10$ and $L = 5$, throughout this figure and the end time is $T_{end} = 2000$. In the left and middle column, the alignment ordering function is plotted for different values of the repulsive strength parameter, b, for LVM (left) and HVM (middle). In the right column, the alignment order parameter C_0 is shown. Note that in plots (a,b,d,e) the bars are plus/minus the standard error, while in plots (c,f) the bars give the range corresponding to the confidence intervals generated in calculating the order parameters, as detailed in Method Section.

III. SUPPLEMENTARY MOVIES

Supplementary Movie 1

Evolution of the collection of the model LVM sperm cells obtained from the same simulation as the snapshot shown in Fig. 3(a) of the main text.

Supplementary Movie 2

Evolution of the collection of the model HVM sperm cells obtained from the same simulation as the snapshot shown in Fig. 3(b) of the main text.

[1] J. M. Cummins and P. F. Woodall, *On mammalian sperm dimensions*, J. Reprod. Fert., 75 (1985) 153-175.

[2] H. Gadêlha, H., E. A. Gaffney, D.J. Smith and J.C. Kirkman-Brown *Nonlinear instability in flagellar dynamics: a novel modulation mechanism in sperm migration?*, J. Roy. Soc. Interface, 7 (2010) 1689-1697.

[3] K. Ishimoto and E. A. Gaffney, *Boundary element methods for particles and microswimmers in a linear viscoelastic fluid*, J. Fluid Mech., 831 (2017) 228-251.

[4] K. Ishimoto, H. Gadêlha, E. A. Gaffney, D. J. Smith and J. Kirkman-Brown, *Coarse-graining the flow around a human sperm*, Phys. Rev. Lett., 118 (2017) 124501.

[5] H. Moore, K. Dvorakova, N. Jenkins and W. Breed, *Exceptional sperm cooperation in the wood mouse*, Nature, 418 (2002), 174-177.

[6] Rothschild, *Non-random Distribution of Bull Spermatozoa in a Drop of Sperm Suspension*, Nature 198 (1963),1221-1222.

[7] D. J. Smith, E. A. Gaffney, J. R. Blake and J. C. Kirkman-Brown, *Human sperm accumulation near surfaces: a simulation study*, J. Fluid Mech., 621 (2009) 289-320.

[8] C. K. Tung, C. Lin, B. Harvey, A. Fiore, F. Ardori, M. Wu and S. S. Suarez, *Fluid viscoelasticity promotes collective swimming of sperm* Sci. Rep., 7 (2017), 3152
